# Integrity and trust in anesthesiology research: recognizing and combating scientific misconduct

**DOI:** 10.1016/j.bjane.2026.844843

**Published:** 2026-10-11

**Authors:** André P. Schmidt, Federico Bilotta, Maria José C. Carmona, Liana M.T.A. Azi

**Affiliations:** a Universidade Federal do Rio Grande do Sul (UFRGS), Hospital de Clínicas de Porto Alegre (HCPA), Department of Anesthesia and Perioperative Medicine, Porto Alegre, RS, Brazil; b Santa Casa de Porto Alegre, Department of Anesthesia, Porto Alegre, RS, Brazil; c Hospital Nossa Senhora da Conceição (GHC), Department of Anesthesia, Porto Alegre, RS, Brazil; d Universidade Federal do Rio Grande do Sul (UFRGS), Programa de Pós-graduação em Ciências Cirúrgicas, Porto Alegre, RS, Brazil; e Universidade Federal do Rio Grande do Sul (UFRGS), Programa de Pós-graduação em Ciências Pneumológicas, Porto Alegre, RS, Brazil; f Faculdade de Medicina da Universidade de São Paulo (FMUSP), Programa de Pós-Graduação em Anestesiologia, Ciências Cirúrgicas e Medicina Perioperatória, São Paulo, SP, Brazil; g “Tor Vergata” University of Rome, Department of Anesthesiology and Intensive Care, Rome, Italy; h Faculdade de Medicina da Universidade de São Paulo (FMUSP), Discipline of Anesthesiology, São Paulo, SP, Brazil; i Universidade Federal da Bahia (UFBA), Hospital Universitário Professor Edgard Santos, Department of Anesthesiology, Salvador, BA, Brazil

Biomedical research is a cornerstone of clinical advancement, providing the evidence that underpins modern medical practice. That enterprise rests on trust. Readers accept a paper because they expect that the participants existed, the data were collected, and the analysis was reported as planned. When an article is retracted for fabrication, falsification, or plagiarism, the damage is not confined to one citation. The retraction undermines confidence in the surrounding literature and, if the record is left uncorrected, in the credibility of science itself. The consequences are concrete: misleading clinical practice, jeopardized patient safety, wasted resources, and eroded public trust.^1^^,^^2^ The medical community must remain vigilant in an era of heightened scrutiny and “publish or perish” pressures. Recognizing, preventing, and addressing scientific misconduct is a collective responsibility to protect scientific credibility and patient welfare.

Research misconduct and related failures are not of equal gravity, and they should not be handled as if they were. Research misconduct, in the narrow sense of fabrication, falsification, and plagiarism, is intentional deception. It is grounds for retraction, institutional sanction, and, in some jurisdictions, professional or legal penalty. Questionable research practices sit below that threshold but still distort the record: p-hacking, selective reporting, inadequate blinding, outcome switching, and hypothesizing after the results are known. They are not always prosecutable as fraud, yet they inflate false-positive findings and should be treated as integrity problems, not as style. Methodological weakness is a third category. Inadequate blinding, an underpowered comparison, or a poorly defined endpoint can make a study unusable without anyone having invented a number. Honest error is a fourth. A mislabeled table or a coding mistake calls for a correction, not a misconduct finding, as long as it is disclosed and fixed. All of them can waste funding, distort guidelines, and feed irreproducible findings. Retracted papers, whether withdrawn for misconduct or left uncorrected after a fatal flaw, can linger as “zombie” citations and continue to influence subsequent research and meta-analyses.^3^^,^^4^ In anesthesiology and perioperative medicine, the stake is clinical. Tainted evidence has supported protocols later judged unsafe, which is why appraisal of data integrity belongs in specialty training alongside appraisal of design.

Scientific misconduct is not confined to any single specialty but has left a notable imprint on clinical anesthesiology and perioperative medicine. Several high-profile cases have underscored the devastating effects of misconduct in our field. For instance, anesthesiologist Scott Reuben fabricated data in at least 21 papers between 1996 and 2008, inventing patient outcomes to support pain management protocols involving drugs like Rofecoxib and Celecoxib.^3^ This led to multiple retractions, a six-month prison sentence for healthcare fraud, and a reevaluation of multimodal analgesia guidelines that had influenced clinical practice for years.^3^ Similarly, Joachim Boldt’s fabrication across studies of hydroxyethyl starch for fluid management remains one of the largest retraction scandals in medical history.^4^ By mid-2026, his retraction count had reached more than 200, and journals were still removing papers more than a decade after the original institutional findings, including articles previously left standing.^5^ Some of those papers continued to be cited years after the scandal became public, illustrating how slowly the scientific record self-corrects even when fraud is no longer in dispute.^5^ Notably, some of the forged papers on hydroxyethyl starches supported clinical practices that were subsequently shown to threaten patient safety.^6^^,^^7^ These incidents underscore the direct harm to patient outcomes, as falsified data led to the adoption of potentially dangerous protocols in perioperative settings, necessitating urgent corrections to clinical guidelines and practices.

The case of Yoshitaka Fujii, an anesthesiologist who fabricated data in more than 160 papers on postoperative nausea and vomiting over two decades, has also exposed flaws in peer review systems. Statistical inconsistencies in his results went undetected for years, leading to revisions of antiemetic guidelines worldwide and underscoring the need for enhanced detection tools to support post-publication scrutiny.^8^ A more recent specialty-specific scandal involves Hironobu Ueshima, formerly of Showa University Hospital in Tokyo. Institutional and Japanese Society of Anesthesiologists investigations concluded that 142 of his articles, including original papers, case reports, and a large volume of letters on regional anesthesia and plane blocks, contained fabricated or falsified data and/or improper authorship.^9^ More than 100 of those papers were subsequently retracted, placing Ueshima among the most-retracted authors in the biomedical literature and contaminating a body of work that had been rapidly absorbed into regional-anesthesia practice.^9^ The scale of these cases has led the specialty in Japan to treat prevention as a society-level responsibility, including mandatory ethics training, centralized data stewardship, and confidential reporting pathways.^10^ They also show that fraud is not confined to large multicenter trials: high-volume letters and “technique” papers, once absorbed into everyday practice, can contaminate the clinical literature just as efficiently.

Another egregious example involves cardiologist Don Poldermans, whose falsified data in the DECREASE trials on beta-blockers for non-cardiac surgery patients infiltrated European clinical guidelines.^11^ Dismissed from his institution in 2011, Poldermans’ work resulted in over 10 retractions and forced revisions to the European Society of Cardiology's perioperative care recommendations, demonstrating how fraudulent research can misdirect treatment protocols on a continental scale and endanger patient safety. Investigations uncovered not only data fabrication but also ethical lapses, such as unreported adverse events and conflicts of interest with pharmaceutical sponsors. The fallout extended beyond retractions: a meta-analysis^12^ excluding his work showed an increased mortality rate associated with beta-blockers, prompting revisions to guidelines by the European Society of Cardiology and the American College of Cardiology.

A comprehensive analysis of retraction processes for papers by Boldt, Reuben, and Fujii highlights ongoing deficiencies in journal and publisher responses.^13^ By mid-2018, 94% of the 313 eligible papers had been retracted. Still, median retraction times varied significantly (1 month for Reuben, 4 months for Boldt, and 13 months for Fujii), with delays attributed to the larger number of affected journals and a lack of coordinated notification. Only 85% of retraction notices complied with the Committee on Publication Ethics (COPE) guidelines, and 21% of retracted articles lacked transparent watermarks to alert readers.^13^ The study's inquiries prompted 16 additional retractions, revealing that many journals were unaware of the need to act or cited legal concerns, emphasizing the necessity for standardized, proactive mechanisms to swiftly remove fraudulent work from the scientific record and minimize its lingering impact on research and clinical practice.

These cases not only undermined specific therapeutic recommendations but also exposed systematic vulnerabilities in editorial oversight, peer review, and institutional controls. A seminal statistical analysis by J. B. Carlisle in 2012^8^ scrutinized 168 randomized controlled trials published by Yoshitaka Fujii from 1991 to July 2011, evaluating data integrity by comparing reported distributions of categorical and continuous variables with those expected from random sampling. Employing chi-squared goodness-of-fit tests and Monte Carlo simulations, the study found that 85% of variable distributions were inconsistent with chance expectations, with probabilities ranging from 1 in 25 to less than 1 in 10^33^. In 141 human studies, all 13 continuous variables and 7 of 11 categorical variables deviated significantly; individually, 96 of 134 randomized studies showed inconsistencies, compared to only 12 of 139 in trials by other authors. These extreme deviations indicate non-random data patterns, likely due to fabrication, underscoring the need to exclude Fujii's work from scientific evidence and highlighting the role of statistical tools in uncovering fraud.

Fraud is not confined to clinical research. Experimental sciences, particularly in biology, are equally susceptible. In a recent perspective, Dubiner and Arbel-Groissman^14^ emphasized the alarming ease with which raw data from common laboratory devices, such as microscopes, gel blots, imaging systems, microplate readers, and spectrophotometers, can be fabricated or manipulated without detection, often through editable output files like spreadsheets or images. Their study noted undoubtedly false data (duplicated or impossible values) during peer review, underscoring that the effort to generate and publish fraudulent results is minimal compared to detection. Positive result bias further compounds the problem, as negative findings are rarely published, creating fertile ground for selective reporting, misconduct, and a “secret third option” to the publish-or-perish dilemma: “simply cheat”. Conducting clinical and experimental research presents formidable challenges, including securing funding, recruiting suitable participants, navigating ethical approvals, and dealing with the inherent variability in biological responses that often yields inconclusive or negative results for specific interventions. These difficulties can tempt researchers into misconduct, such as data fabrication or selective reporting, to produce “positive” outcomes that satisfy publication demands and career-advancement pressures in a competitive academic environment. Reasons for producing fabricated data encompass academic pressures, where career progression hinges on publication metrics; financial incentives from grants or industry ties that favor positive results; personal ambitions for recognition; institutional demands for productivity; and a lack of robust oversight, all of which can drive researchers to invent outcomes rather than report genuine, often negative, findings. Additionally, funding agencies bear significant responsibility for mitigating fraud, as their emphasis on high-productivity metrics, such as publication counts and grant deliverables, can inadvertently exacerbate pressures that lead to misconduct. To counter this, agencies should revise evaluation criteria to prioritize research quality, reproducibility, and ethical compliance over sheer output, while incorporating integrity audits into grant reviews and providing resources for ethics training.

A recent investigation into a specific open-access journal reveals stark anomalies in editorial handling, where a small subset of editors, comprising just 0.25% of the total, oversaw the publication of 30.2% of retracted articles, despite editing only 1.3% of the journal's overall output.^15^ This disproportionate involvement, coupled with networks of reciprocal authorship and editing among flagged individuals from multiple countries, underscores the coordinated nature of fraud facilitation. Such patterns not only evade random editor assignment but also highlight how interconnected groups exploit journal processes, leading to retractions that often cite concerns over authorship, competing interests, and peer-review integrity.

Efforts to detect and prevent research misconduct have evolved but remain insufficient. Journals have adopted stricter protocols, including mandatory clinical trial registration, professional editorial offices with comprehensive statistical review, comprehensive guidelines for editors and peer reviewers, and the systematic use of plagiarism detection software.^16^ However, as shown by Fiore et al.^17^ in their 2021 scoping review analyzing 423 retracted publications in anesthesiology, the risk of retraction for fraud does not differ significantly across journals of different impact factors or quartiles. These findings reveal that high-profile journals are not immune, underscoring the need for universal and continuous vigilance across all tiers to address systemic vulnerabilities. Despite the increasing number of retractions, it is crucial to emphasize that continuing medical education and evidence-based clinical practice still rely heavily on regular literature reading. Clinicians must be trained to appraise sources critically, cross-reference with retraction databases, and integrate updated guidelines to maintain high standards of care, ensuring that retractions do not deter engagement with the evolving body of evidence but instead foster a more discerning approach to its application.

A recent review by Nato and Bilotta^18^ further illuminates these challenges, reporting over 10,000 paper retractions in 2023, with perioperative medicine heavily impacted. The review highlights high-profile cases in which forged data infiltrated guidelines, leading to suboptimal care and patient harm. The authors propose leveraging machine learning and artificial intelligence (AI) for pre-publication screening, alongside tools like the REAPPRAISED checklist^19^ and Benford's Law,^20^ to detect anomalies and enhance peer review. Integrating databases such as Retraction Watch with Scopus and Zotero is also suggested to exclude retracted papers from reviews, reinforcing the need for robust, technology-driven safeguards to protect research integrity. Importantly, excluding retracted papers from a review is not a software preference, and it is not a task to leave to the reader after publication. It is an author’s duty, enforced by the journal at revision and editorial analysis.

Platforms like Retraction Watch play a pivotal role in exposing fraudulent researchers by maintaining a comprehensive, publicly accessible database of retractions, which tracks misconduct, plagiarism, and errors across disciplines, including anesthesiology.^21^ By highlighting patterns, such as disproportionate retractions from paper mills or individual authors, and advocating for transparency, Retraction Watch not only accelerates the removal of flawed studies but also deters future fraud through public accountability, often prompting investigations that lead to additional retractions and institutional actions. Similar strategies, including whistleblower protections and collaborative publisher alerts, amplify this impact by fostering a culture of vigilance; for example, by revealing how publication pressures fuel retractions and ensuring “zombie papers” are flagged in citations, these tools help restore trust in science while underscoring the need for proactive, coordinated responses to misconduct. Independent organizations like Retraction Watch and PubPeer, supported by regional legislation, are essential for monitoring and exposing misconduct. Advocacy is needed for actual punishments, including criminal charges, license revocations, and institutional sanctions, to create deterrents beyond mere retractions and ensure accountability. Whistleblowing plays a crucial role in uncovering misconduct, yet many researchers hesitate because of fears of retaliation. Institutions must implement robust protection mechanisms, including anonymous reporting channels and legal safeguards, to encourage disclosures without professional repercussions. One practical suggestion is to add a standardized question at the end of each downloadable article, or in a dedicated channel on the journal’s site, asking readers whether they have any concerns or suspicions about integrity issues in any publication, to support proactive community vigilance and streamline investigations.

The rise of paper mills (clandestine operations that fabricate manuscripts *en masse* and sell authorship) has become a profitable industry, infiltrating even reputable journals and undermining scientific credibility. As detailed by Candal-Pedreira et al.,^22^ an analysis of 416 retracted mill-originated papers from 2022 uncovered dense networks where these fraudulent works cite and are cited by other retracted papers, forming clusters of mutually interconnected retractions and a broader web of papers, highlighting citation manipulation as a detection signal and evasion tactic. Parker et al.^23^ further elucidate the operational evolution, from template-based fabrication and fake reviewer infiltration to editorial board compromises, estimating mills now comprise over 1.5% of the literature, with AI exacerbating production and obfuscation. Recent escalations underscore the urgency: in 2023-2025, publishers like Hindawi retracted over 11,000 mill papers, Sage withdrew hundreds, and Wiley paused special issues after detecting mill activity in one-in-seven submissions, while investigations revealed mills bribing editors and hijacking journals.^23^ The industrial scale of this activity is now measurable. A 2026 machine-learning screen of 2.65 million cancer papers flagged 9.87% as textually similar to known paper-mill products, with the signal rising over time and appearing even in journals in the top decile by impact factor.^24^ Flagged papers also attracted more citations than apparently genuine work in the same field, which can inflate journal metrics and recycle fraudulent claims through systematic reviews. These findings do not prove that every flagged article is fabricated, but they show that mills are no longer confined to obscure outlets and that textual and citation forensics must become routine editorial tools, including in perioperative journals. Recent examples in Brazil highlight the sale of authorship positions and paper mill operations, which facilitated fraudulent publications and led to retractions in SciELO-indexed journals.^25^ Investigations reveal networks selling slots in fabricated manuscripts, often infiltrating reputable outlets.^26^ The lack of effective measures, such as nationwide AI-driven detection systems or mandatory authorship audits, impedes control, allowing these practices to persist amid limited resources and enforcement.

To combat the rising threat of organized fraud via paper mills, which has shown exponential growth in retractions and fabricated products, institutions must prioritize prevention through ethics training and strict data-integrity policies. The COPE guidelines^27^ serve as a critical tool in this effort, providing standardized frameworks for publishers, editors, and researchers to address misconduct transparently and consistently. These guidelines outline best practices for handling allegations of fraud, ensuring timely retractions, and promoting ethical publishing standards, thereby acting as a vital ally in safeguarding research integrity. Complementary tools like plagiarism detectors (e.g., iThenticate, Turnitin), statistical anomaly checks, and open science initiatives for data sharing further fortify defenses, ensuring that integrity is valued over publication metrics.^28^ While similarity detection software effectively identifies textual plagiarism through similarity checks, it falls short in detecting more sophisticated forms of fraud, including data fabrication, image manipulation, or statistical anomalies. This limitation underscores the need for complementary tools, such as statistical audits and image forensics, to provide a more comprehensive safeguard against misconduct. In this context, editors and reviewers serve as frontline defenders of research integrity, responsible for detecting anomalies in methodology, data, and ethics during peer review. Their actions include requiring conflict-of-interest disclosures, using plagiarism checkers, requesting raw data for verification, and applying statistical assessments to identify irregularities. When misconduct is suspected, editors must initiate investigations per COPE protocols, issue expressions of concern, and execute timely retractions, while reviewers should flag image manipulations or implausible results, fostering a collaborative effort to uphold standards.^27^ The 2025 revision of the COPE retraction guidelines makes that duty more explicit. Editors may retract when they no longer have confidence in the findings, including for paper-mill activity, identity theft or fictitious authorship, and undisclosed involvement of artificial intelligence; the guidance also endorses batch retractions when systematic manipulation of the publication process is identified, rather than waiting for article-by-article institutional verdicts.^27^ COPE’s 2026 relaunch of its paper-mills working group, together with the multi-stakeholder United2Act initiative, further recognizes that coordinated, AI-amplified fraud now outpaces case-by-case investigation.^29^ Journals in anesthesiology and perioperative medicine should align their instructions to authors and their retraction workflows with this updated standard.

The model of access, gold versus diamond open access, may play a critical role in influencing integrity. In gold open access, authors pay an article processing charge. Many gold journals keep a rigorous review process and contribute to dissemination. The same fee, however, can reward volume. Some profit-driven outlets have weakened review, and retraction rates are higher in parts of that market, especially where predatory titles exploit authors in low-resource settings.^30^ Diamond open access removes the fee for authors and readers and is usually supported by a society, an institution, or a public fund. That has clear advantages for access and for authors who cannot pay. It does not, by itself, lower the risk of misconduct. Integrity in either model depends on editorial governance: independent editors, a transparent review process, the ability to reject weak or suspicious work, and a retraction policy that is actually used. A diamond journal with a captured editorial board is not safer than a gold journal with a functioning one. The useful question is not which label the journal carries, but who controls acceptance and who can be required to correct the record. Nevertheless, while neither model is immune to abuse and both require ongoing vigilance to prevent it, transitioning to broader adoption of diamond open access could enhance research integrity by democratizing access without the pitfalls of pay-to-publish systems.

The struggle to ensure controlled, high-quality literature continues, with fraudulent publishing activities posing significant challenges despite advances in detection. A recent publication highlights innovative approaches to combat this, such as red-flagging methods that identify the mass production of fake publications in biomedical science using indicators such as unnatural citation patterns and textual anomalies.^31^ Open science initiatives, which emphasize transparency through data sharing, preregistration, and accessible repositories, can mitigate research misconduct by enabling independent verification and reducing selective reporting.^32^ Beyond access models, open science encompasses key pillars such as the FAIR (Findable, Accessible, Interoperable, Reusable) principles for data sharing, which ensure that datasets are easily discoverable and usable by others, thereby enhancing verification and collaboration.^33^ Additionally, open peer review, where reviewer identities and comments are disclosed, and the sharing of analysis codes create a comprehensive ecosystem that promotes reproducibility and transparency, potentially inhibiting misconduct by allowing broader scrutiny and reducing opportunities for hidden manipulations. In this framework, readers play a vital role as post-publication reviewers, contributing through platforms like PubPeer to identify errors or inconsistencies after publication, thereby extending the peer review process and strengthening community-driven accountability.

Traditional strategies for enhancing research integrity, such as prospective trial registration on platforms like ClinicalTrials.gov, adherence to CONSORT guidelines for transparent reporting, and compliance with PRISMA for systematic reviews, suffer from inconsistent implementation. Many trials remain unregistered, allowing outcome switching and publication bias to thrive. At the same time, non-adherence to guidelines obscures methodological flaws, enabling misconduct to evade detection and underscoring the gap between policy and practice. Registered reports represent a particularly potent preventive tool against publication bias and p-hacking, as this format involves peer review and in-principle acceptance of the study methodology and analysis plan before data collection, ensuring that results are published regardless of outcome and reducing the incentive for *post hoc* manipulations. By shifting the emphasis from results to rigorous design, prospective registration of studies promotes greater transparency and reproducibility in research, with growing adoption demonstrating its efficacy in curbing questionable practices.

Research misconduct that has already entered the perioperative literature will not leave unless the workflow that admitted it changes. Moving forward, several additional strategies may help protect research integrity in anesthesiology and beyond:^1^^,^^28^^,^^32^^,^^34^ i. Retraction check – Authors of reviews and other evidence syntheses must search the cited record, state that no retractions were found or that a specific number were removed from the analysis; ii. Plagiarism screening – Every submission should be run through similarity software, such as iThenticate, before review; iii. Bibliographic authentication – Each cited title, year, and identifier should resolve in PubMed or Crossref before acceptance, because fabricated references may pass conventional review; iv. Pre-publication integrity screening – Statistical anomaly checks, image forensics, and data forensics may be considered routine in peer review, alongside tools such as the REAPPRAISED checklist; v. Raw data on request – Authors should deposit or supply data in a form that preserves the original export and records later changes, so reviewers can separate honest error from fabrication; vi. Authorship that can be defended – Gift, guest, and sold authorship should be grounds for rejection; every listed author must meet a stated contribution standard; vii. Limited use of artificial intelligence – Models may assist screening for anomalies. They may not invent text, data, images, or references, and any use must be disclosed; viii. Negative results – Well-conducted negative studies should be publishable, so a null finding is not an incentive to switch outcomes or fabricate a positive one; ix. Institutional accountability – Departments and funders should audit high-volume output, and an independent body should be able to investigate when the host institution is conflicted; x. Education and culture change – Widespread and ongoing training in research ethics, transparency, and reproducibility is essential. Cultivating a culture that prioritizes research quality over quantity remains a critical long-term goal. Ethics and data-handling courses should be required for residents and graduate students, and professional societies, such as the Brazilian Society of Anesthesiology, could lead training in high-risk areas like statistical analysis and ethical data handling to deter misconduct.

While AI holds promise for detecting fraud through anomaly screening and enhanced peer review,^34^ it also exacerbates misconduct by enabling the rapid fabrication of deceptive content. Generative AI models, such as large language models, can produce plausible but entirely invented research papers, datasets, images, and even statistical analyses, lowering the barrier for fraudsters to create “paper mill” outputs at scale.^35^ A quieter but rapidly growing marker of the same problem is the fabricated citation. An audit of 2.5 million biomedical papers in PubMed Central found 4,046 references whose titles matched no existing publication, across 2,810 articles. The rate rose more than twelve-fold between 2023 and early 2026, reaching one in 277 papers in the first weeks of 2026, a trajectory that tracks the publication lag after widespread adoption of large language models.^36^ Because hallucinated references are often correctly formatted and attributed to real authors, conventional peer review rarely catches them. For anesthesiology editors, automated reference verification should therefore sit alongside plagiarism and image-forensics checks at submission, and authors should be required to attest that they confirmed every citation against a bibliographic database. For instance, AI-driven tools have been implicated in generating fraudulent medical articles that mimic authentic research, leading to undetected plagiarism, misinformation, and manipulated results that impair scientific credibility.^35^ The same tools that can screen for anomalies can also produce the material being screened. The risk that matters here is not generic misuse of AI. It is the fabricated paper, the invented dataset, the manipulated image, and the citation that points to no article. Anesthesiology journals need AI-specific forensics for those products, and instructions to authors that say what use of a model must be disclosed.

A clear research agenda for anesthesiology may include systematic studies on fraud detection methods, such as the application of statistical tools like Benford's Law for identifying anomalies in reported data from anesthesia trials, as demonstrated in analyses of falsified papers where deviations from expected digit distributions have proven effective in uncovering fabrication.^18^ Additionally, it should explore the impact of retracted studies on clinical practice, examining how “zombie papers” persist in citations within systematic reviews and guidelines, leading to misguided perioperative protocols and potential patient harm, with evidence showing that retracted randomized controlled trials continue to be inappropriately referenced despite clear retraction notices. Furthermore, the agenda ought to prioritize the development of tools to track research integrity indicators prospectively, including automated platforms for evaluating adherence to good clinical practices in randomized controlled trials, or integrated databases that monitor retraction patterns and citation networks to preempt the dissemination of flawed evidence in anesthesiology literature. To broaden its scope, this agenda could incorporate evaluations of AI's dual role in fraud detection and perpetuation, investigating how AI-driven anomaly screening enhances peer review while also enabling sophisticated data fabrication in paper mills, necessitating the creation of AI-specific ethical forensics tailored to high-stakes fields like anesthesia and perioperative medicine. It should also assess the effects of open science practices on reducing misconduct, such as through preregistration and data sharing that promote transparency and hinder selective reporting, with studies indicating that these initiatives foster a culture of accountability and decrease the incidence of questionable research behaviors in biomedical fields. Moreover, research on the prevalence and consequences of predatory publishing in anesthesiology is essential, given exploitative models that prioritize volume over rigor, leading to higher retraction rates and the infiltration of low-quality studies into clinical decision-making. Finally, the agenda must evaluate the efficacy of ethical training programs in preventing scientific fraud, including mandatory responsible conduct of research workshops that emphasize transparency and institutional accountability, as evidence suggests these interventions can significantly mitigate misconduct when integrated early in researchers' careers.^37^ Ethical training should be integrated into the curricula of medical residents and graduate students, emphasizing responsible conduct of research, data integrity, and the consequences of misconduct to raise a generation of clinicians and researchers committed to upholding scientific standards from the outset of their careers.

National postgraduate program evaluations have traditionally been heavily reliant on publication metrics, such as the number of papers and their impact factors, which inadvertently fuel publication pressures and contribute to scientific misconduct. To address these vulnerabilities, adaptations are essential to incorporate broader, more holistic criteria that value quality, societal relevance, and ethical practices over volume of publications. In Brazil, the Coordination for the Improvement of Higher Education Personnel (CAPES), a federal agency that coordinates efforts to improve the quality of Brazilian faculty and postgraduate students, has implemented a transformative evaluation system for postgraduate programs *stricto sensu* during the 2025–2028 cycle assessment.^38^ This system adopts a multidimensional approach that integrates bibliometric indicators with qualitative evaluations of societal impact, open science practices, diversity, inclusion, and alignment with United Nations Sustainable Development Goals, guided by principles of peer review, comparability, retrospective analysis, classification, collaboration, and transparency to ensure community involvement and holistic assessment. A further change is that, for this cycle, individual articles, not only the journals that publish them, are classified, combining venue metrics with article-level quality, societal impact, and open-science practices.^38^ This shift is intended to weaken the incentive to chase journal strata regardless of the integrity of the underlying work. Future assessments in Brazil will be crucial to determine the effectiveness of these new strategies in reducing misconduct and promoting sustainable research cultures, offering valuable insights that could inspire similar regulatory changes in other countries grappling with comparable challenges in postgraduate education.

In conclusion, biomedical research is fundamental to improving patient care, but its foundation must be built on integrity. In anesthesiology and perioperative medicine, where clinical decisions often carry high stakes, the need to recognize, prevent, and address scientific misconduct is especially urgent. Safeguarding the scientific record is not merely a technical task, it is a moral imperative shared by researchers, reviewers, editors, and institutions alike. Reaffirming our commitment to transparency, honesty, and rigorous science is essential to ensuring the sustainable advancement of medical knowledge and the continued trust of the society we serve.

## Conflicts of interest

The authors declare no conflicts of interest.

## Data Availability

No new data were created or analyzed in this study. Data sharing is not applicable to this article.
